# An Infant Prodigy

**Published:** 1890-11

**Authors:** 


### An Infant Prodigy.

Dr. S. V. Clevenger gives (*Alien. and Neurol.*) an acconut of the wonderful memory of a three year old child, Oscar Moore. Oscar is a colored boy who was born blind. He has mastered an appalling array of statistics, such as the areas in square miles of hundreds of countries, the population of the world's principal cities, the birthdays of the presidents, names of the cities of the United States, etc. His mental development seems to be symmetrical, and he desires to understand the meaning of all that he is taught. Of the seven brothers and sisters of Oscar all of them died probably of tuberculosis, excepting his two sisters, who are living. While idiots sometimes show a wonderful memory in certain lines, there is no reason to suppose that Oscar is in any degree idiotic. One of the conspicuous faculties of many great men is a wonderful memory.

A CERTAIN very rich and avaricious man conceived the idea of receiving gratuitous advice from the English doctor Abernethy. For this purpose he invited the physician to a dinner party, and in the course of ordinary conversation, he insinuated his own case by remarking:—"Doctor, suppose a man has symptoms—thus and thus. What would you advise him to take?" Abernethy answered: "I should advise him to take medical advice."—*Edgar Poe.*

